# Transcriptomic Analysis of Avocado Hass (*Persea americana* Mill) in the Interaction System Fruit-Chitosan-*Colletotrichum*

**DOI:** 10.3389/fpls.2017.00956

**Published:** 2017-06-08

**Authors:** Luis-Ángel Xoca-Orozco, Esther Angélica Cuellar-Torres, Sandra González-Morales, Porfirio Gutiérrez-Martínez, Ulises López-García, Luis Herrera-Estrella, Julio Vega-Arreguín, Alejandra Chacón-López

**Affiliations:** ^1^Laboratorio Integral de Investigación en Alimentos, Instituto Tecnológico de TepicTepic, Mexico; ^2^Laboratorio Nacional de Genómica Para la Biodiversidad, Center for Research and Advanced Studies of the National Polytechnic Institute (CINVESTAV)Guanajuato, Mexico; ^3^Laboratorio de Ciencias AgroGenómicas, Escuela Nacional de Estudios Superiores, Universidad Nacional Autónoma de MéxicoGuanajuato, Mexico

**Keywords:** avocado Hass, *C. gloeosporioides*, RNA-seq, chitosan, elicitor, resistance

## Abstract

Avocado (*Persea americana*) is one of the most important crops in Mexico as it is the main producer, consumer, and exporter of avocado fruit in the world. However, successful avocado commercialization is often reduced by large postharvest losses due to *Colletotrichum* sp., the causal agent of anthracnose. Chitosan is known to have a direct antifungal effect and acts also as an elicitor capable of stimulating a defense response in plants. However, there is little information regarding the genes that are either activated or repressed in fruits treated with chitosan. The aim of this study was to identify by RNA-seq the genes differentially regulated by the action of low molecular weight chitosan in the avocado-chitosan-*Colletotrichum* interaction system. The samples for RNA-seq were obtained from fruits treated with chitosan, fruits inoculated with *Colletotrichum* and fruits both treated with chitosan and inoculated with the fungus. Non-treated and non-inoculated fruits were also analyzed. Expression profiles showed that in short times, the fruit-chitosan system presented a greater number of differentially expressed genes, compared to the fruit-pathogen system. Gene Ontology analysis of differentially expressed genes showed a large number of metabolic processes regulated by chitosan, including those preventing the spread of *Colletotrichum*. It was also found that there is a high correlation between the expression of genes *in silico* and qPCR of several genes involved in different metabolic pathways.

## Introduction

The avocado (*Persea americana*) cv Hass, is from the economic point of view, one of the most important fruits in Mexico because it is the main producer, exporter and consumer of this fruit in the world. However post-harvest diseases represent a serious problem for exports of avocado fruit. Anthracnose, caused by *Colletotrichum* sp., is one of the most economically important diseases of the avocado fruit during storage and marketing, with losses close to 20% of the total avocado production (Freeman et al., 1996; Rodríguez-López et al., 2009). To control this pathogen, chemical fungicides are normally used, even though they have the potential of affecting the environment and the health of consumers (Brent and Hollomon, 2007). For this reason it is important to seek biological alternatives to the use of fungicides, such as chitosan, which has no polluting characteristics and acts as an elicitor of plant defense responses. Chitosan, or poly [ß- (1-4) -2-amino-2-deoxy-D-glucopyranose], is a copolymer of N-acetyl-D-glucosamine units derivative from chitin by a deacetylation treatment in alkaline medium (Islam et al., 2011). The elicitor activity of chitosan is well documented, for instance, it has been reported that upon external application of chitosan the production of chitinases, protease inhibitors and phytoalexins can be induced, triggering a defense response in plants (Terry and Joyce, 2004; Amborabé et al., 2008). However, the mechanism by which chitosan elicits a defense response in plants is not fully elucidated, although some reports indicate that secondary metabolites could have an important role in this response. For example, chitosan application in pear (*Pyrus pyrifolia* L. cv. Xuehua) resulted in an increase of phenylalanine ammonia lyase (PAL), polyphenol oxidase (PPO) and peroxidase (POD) activities, as well as in the induction of the expression of β-1,3-glucanase and chitinase genes, which may also be involved in defense against *Alternaria kikuchiana* and *Physalospora piricola* (Meng et al., 2010). Similarly, using a chitosan film in tomato (*Lycopersicon esculentum* Mill) it was observed an increase in the PPO and POD activities, and in the production of phenolic compounds that correlated with an increase of resistance against *Botrytis cinerea* and *Penicillium expansum* (Liu et al., 2007). Moreover, in Arabidopsis seedlings chitosan regulated the expression of defense response genes against *Botrytis cinerea* and the genes involved in biosynthesis of camalexin (Povero et al., 2011). The activity of enzymes like PAL and tyrosine ammonia-lyase (TAL) and the expression of genes like those encoding for PPO, POD and Isoflavone Synthase I and II, have been reported to increase in the presence of chitosan in mango fruit (Khan et al., 2003; Berumen-Varela et al., 2015) and soybean (Chen et al., 2009). More recently, RNA-seq analysis has allowed the identification of defense genes induced in orange leaves upon treatment with chitosan, such as different transcription factors and genes involved in some hormone pathways (Coqueiro et al., 2015). In spite of these previous studies, the mechanism by which the chitosan is perceived by the plant and how it induces resistance remains unknown, therefore it is important to design studies that could help shed some light on these processes.

With the aim of having a better understanding of the mechanisms of action of chitosan in the induction of resistance to *Colletotrichum* sp. in avocado fruit, here we analyzed the global transcriptional profile of avocado fruits treated with chitosan and in the presence or absence of *Colletotrichum*. We also report on the differentially expressed genes in response to chitosan that could be involved in the resistance induced by this elicitor.

## Materials and methods

### Isolation and identification of *Colletotrichum* sp. from avocado fruits

In order to isolate and identify the pathogen causing anthracnose symptoms in avocado, we used fruits that were harvested at physiological maturity stage from an orchard located in Tepic, Mexico. Fungus development was encouraged by placing fruits in chambers at a relative humidity of 90–95% and 25°C for 5 days. Once disease symptoms were developed in the fruit, damaged tissue was washed by immersion in sodium hypochlorite solution (2%) for 2 min, rinsed with sterile water for 2 min and placed on filter paper to eliminate moisture. Moisture-free tissue sections were placed on potato dextrose agar (PDA) (DIBCO) and incubated at 25°C for 24–72 h. Fungus genus was determined according to the taxonomic keys described by Barnett and Hunter (1998) and by molecular techniques using amplification of ITS1 and ITS4 primers according to White et al. (1990).

### Fruit maturity and sampling time

To establish the sampling times for the RNA-seq analysis, we first evaluated the development of anthracnose symptoms caused by *Colletotrichum gloeosporioides* in avocado fruits at different states of maturity: physiological, intermediate and consumer maturity. For this, 10 fruits (for each state of maturity) were inoculated with 4 μL spore suspension (1 × 10^6^ mL^−1^) of *C. gloeosporioides* previously isolated. Development of the infection was monitored in a stereoscope to follow the fruit invasion by the fungus at 0, 1, 2, 6, 9, 24, 72, and 96 h post-inoculation. For RNA-seq experiments we then selected the maturity state “intermediate” and sampling times at 0, 1, 6, 9, and 24 h post-inoculation. In order to study the elicitor effect of chitosan, another group of 10 fruits in a state of “intermediate” maturity were inoculated with 40 μL of the spore suspension and treated with a solution of low molecular weight chitosan (LMWC) 1.5% (w/v) (Sigma Aldrich; viscosity 35 cps in 1% chitosan solution; 96.1% deacetylation), the inoculated/treated fruits were kept at 25°C for 7 days to record the disease development.

### Chitosan treatment

After determining the appropriate sampling conditions for RNA-seq, four treatments were performed: untreated control fruits (C), fruits inoculated with *C. gloeosporioides* (P), fruits treated with LMWC without pathogen (Q) and fruits inoculated with *C. gloeosporioides* and treated with LMWC (QP). Pathogen inoculation (P and QP treatments) was performed using an insulin syringe as follows: 40 μL of a spore suspension (1 × 10^6^ spores/mL) were inoculated by penetrating 3 mm into the fruit peel, C and Q treatments were inoculated with 40 μL of sterile water. After 30 min, fruits were immersed during 1 min into LMWC solution at 1.5% w/v, the treatments that did not contain chitosan (C and P) were immersed in sterile water also for 1 min. Sample collection was performed by cutting sections of 5 × 5 cm of peel and pulp of the avocado fruits, which were immediately frozen in liquid nitrogen and macerated to a fine powder in a sterile mortar. Samples of three biological replicates of each treatment were stored independently as powder at −80°C for later use. For all these treatments samples were taken at: initial time (0 h), early response (1, 6, and 9 h) and late response (24 h). Samples 6 and 9 h were mixed, because no differences in the development of the disease were observed.

### RNA extraction and sequencing

RNA extraction was performed for each of the biological replicates according to the methodology proposed by Djami-Tchatchou and Straker (2012). Then samples from the same treatment were mixed equimolarly. cDNA library preparation and sequencing was carried out at the National Laboratory of Genomics for Biodiversity (LANGEBIO-CINVESTAV-Irapuato, Mexico). Illumina HiSeq 2000 system was used and the sequencing run was 2 × 100 (paired-end). Raw data were obtained as FASTQ files, which include reads and their qualities defined based on PHRED, with an average quality >30. Raw sequencing data is available at NCBI under accession number SUB2518488.

### Bioinformatics analysis

#### Sequence mapping

A reference transcriptome of avocado (*Persea americana* Mill) var drymifolia (TAD) (Ibarra-Laclette et al., 2015) and also a reference transcriptome of avocado fruit (*Persea americana* Mill) cv Hass (TFH) (Kilaru et al., 2015) were used. The Bowtie2 program was used for mapping (Langmead and Salzberg, 2012), this tool also allows us to obtain the read count for any particular gene model. Counting and determination of FPKM (Fragments Per Kilobase of transcript per Million mapped reads) was performed using the program eXpress (Roberts and Pachter, 2013).

#### Statistical analysis

Differentially expressed genes were identified by maximum likelihood analysis (*P* < 0.05) using the R statistical (Foundation for Statistical Computing, 2013) with the EdgeR library (McCarthy et al., 2012). The matrix was compared using information from the control samples (fruit not treated with chitosan and not inoculated at their respective sampling times).

#### Functional annotation

To identify enriched GO terms (Process; Up-regulated and Down-regulated genes) Plant MetGenMAP (Ontology Process, multi-test correction FDR, *p*-value < 0.05) (Joung et al., 2009) as well as Classification SuperViewer (Provart and Tong, 2003) were used. For this analysis we used genes showing highest homology to *A. thaliana* genes, eliminating those locus that were repeated. Likewise, the MapMan program (Usadel et al., 2009) was used to identify genes that are specific of metabolic pathways of interest.

### Gene expression validation

To validate the bioinformatics analysis on gene expression prediction based on the RNA-seq data, we used real-time RT-PCR for quantification of several avocado transcripts. For this, aliquots were taken from the same RNA that was used for sequencing, cDNAs were synthesized from 2 μg of total RNA using the Superscript II (Invitrogen), first strand synthesis procedure was followed by RNase H digestion (Invitrogen, Carlsbad, CA, USA) according to the manufacturer's instructions. Primers for different genes encoding transcription factors, plant disease resistance proteins, genes involved in ethylene biosynthesis and secondary metabolites, as well as a gene as internal control (Table S1) were used. The primers were designed using the Primer Express 2.0 software (Applied Biosystems, Foster City, CA, USA) with melting temperatures (Tm) of 58–60°C, primer lengths of 20–24 bp, and amplicon lengths of 61–150 bp. PCR reactions were performed in 48-well plates in a StepOne™ Real-Time PCR System (Applied Biosystems) using SYBR® Green. Three technical replicates were performed for each evaluation. Reactions were done in 25 μl containing 200 nM of each primer, 5 μl cDNA (corresponding to ~3 ng), and 12.5 μl 2 × SYBR Green Master Mix Reagent (Applied Biosystems). PCR parameters were as recommended by the manufacturer: 95°C for 10 min, 40 cycles of 95°C for 15 s, and 60°C for 1 min. Non-template controls were included for each primer pair and each PCR reaction was completed in triplicate. Dissociation curves for each amplicon were then analyzed to verify the specificity of each amplification reaction; the dissociation curve was obtained by heating the amplicon from 60 to 95°C.

## Results

### Isolation and identification of the pathogen causing anthracnose

*Colletotrichum gloeosporioides* is the causal agent of anthracnose in avocado fruits, whose characteristic is to remain dormant until the fruit ripens to the maturity consumer stage, when the defense system of the fruit is less active, allowing an easier and more effective infection (Rodríguez-López et al., 2009). Symptoms of anthracnose in avocado fruits were characterized as black sunken lesions with salmon-colored dots in the center and white mycelial growth on the surface. Pathogen identification at species level was performed by isolating the fungus from diseased avocado and grown in culture media. Morphological characterization was performed by evaluating mycelial growth at random, white mycelium, cottony little consistency, grayish colony, short hyphae on the edge of growth and yellowing in the bottom of the petri dish. Microscopic analysis showed that the isolate, according to taxonomic keys, belongs to the genus *Colletotrichum* (hyaline conidia, form straight, cylindrical and obtuse at the apex, measuring 9–24 um in length and 3–4.5 um width), while the amplification of ITS1 and ITS4 and subsequent fragment sequencing showed a 98% homology to *C. gloeosporioides* (File S2).

### Determination of ripeness of the fruit for RNA-seq

**Figure 1:**
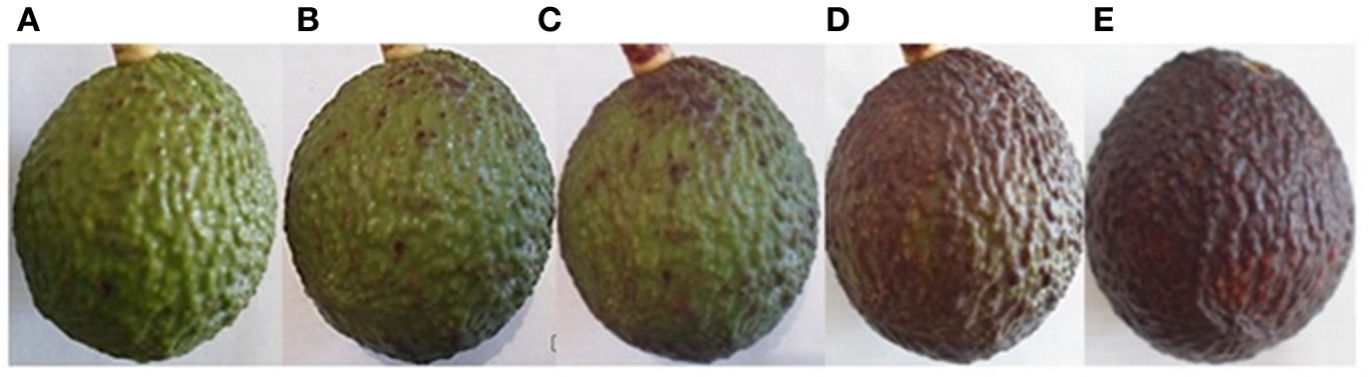
Scale of avocado fruit ripening. **(A,B)** Post-harvest day, 24% dry Matter, firmness > 120 Newton. **(C,D)** Intermediate maturity (between day 5 and 8 post-harvest, 25°C), firmness 60–80 Newton. **(E)** Consumption maturity after 10 days post-harvest, 25°C, firmness < 20 Newton.

To determine the ripeness of the fruit for later transcriptomic analyses, a scale of maturity was established according to the fruit color change from the first postharvest day to maturity consumption stage (Figure 1). During the first 5 days the fruit is in physiological maturity, between 6 and 9 days we considered it as “intermediate maturity,” and after day 10 it is in consumption maturity (stored at 25°C).

**Figure 2:**
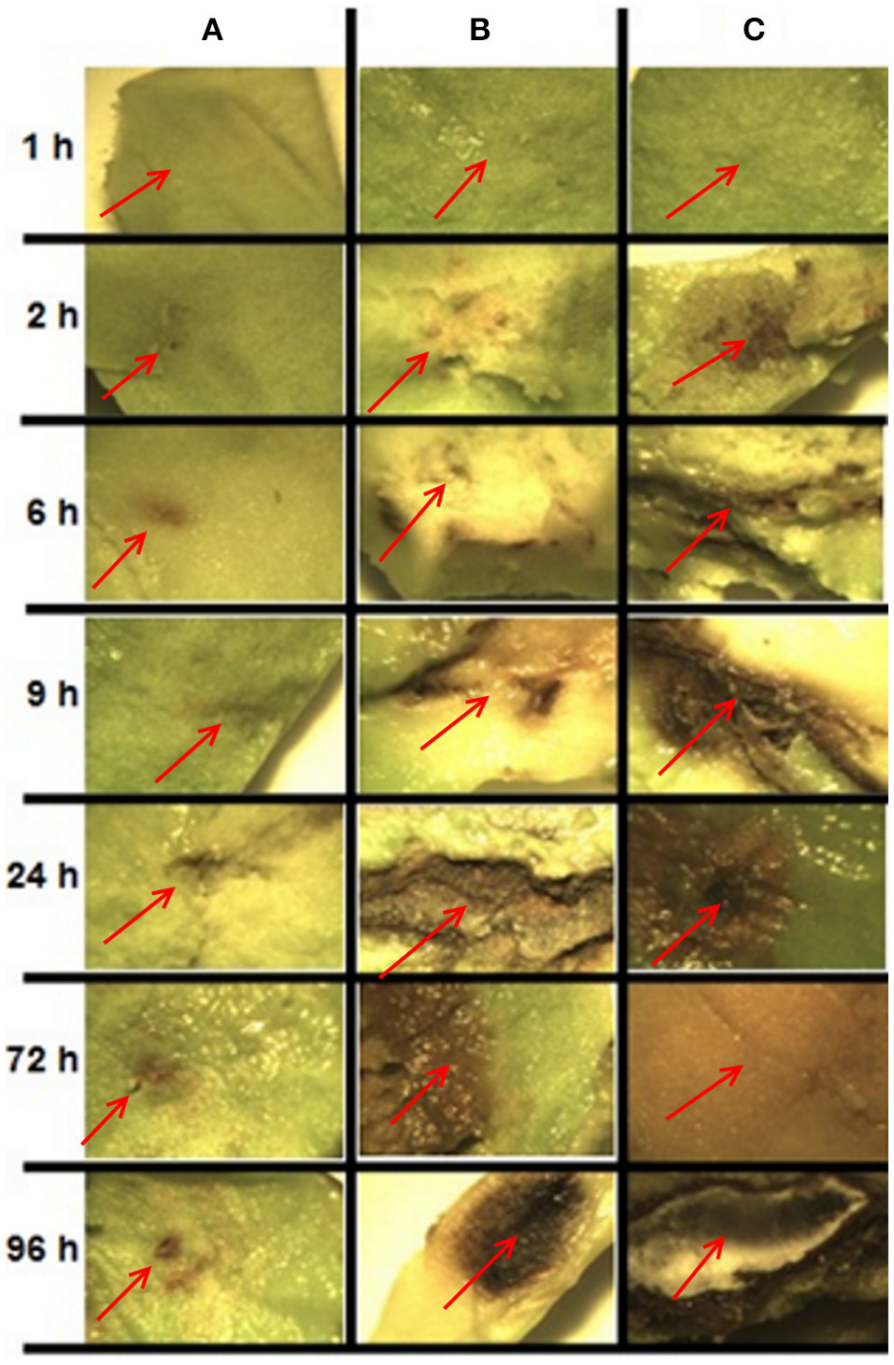
Development of infection caused by inoculation of *C. gloeosporioides* in fruits at different stages of maturity and at different post inoculation times. Each panel shows the pulp of avocado fruit of the area around the inoculation site. **(A)** Physiological maturity, **(B)** intermediate maturity, **(C)** consumption maturity. The red arrow indicates the inoculation site.

In order to determine the sampling times for RNA extraction and sequencing, samples were collected from *C. gloeosporioides* inoculated avocado fruits (without chitosan treatment) at different stages of maturity (physiological, intermediate and consumption) and analyzed for disease symptoms (Figure 2). Inoculated fruits at the physiological maturity stage showed no clear symptoms of pathogen infection, possibly because some antifungal compounds present in the fruit during this stage of maturity prevent fungal infection (Prusky and Lichter, 2007). In fruits at intermediate maturity disease symptoms started to be visible after 9 h of inoculation and at 96 h post-inoculation a limited area of the fruit showed clear disease symptoms. In contrast, in the fruits at the consumption maturity, disease spreads rapidly, becoming visible as early as 2 h post-inoculation, which became widely spread after 96 h post-inoculation. Since the development of infection in fruits at the intermediate maturity stage was slower and allowed to detect more concise changes during the infection process, we decided to use the intermediate fruit ripening stage to investigate the global transcriptional changes induced by chitosan treatment (Q condition), inoculation by *C. gloeosporioides* (P condition) and both, chitosan treatment and pathogen inoculation in the avocado fruits (QP condition).

### Disease reduction in avocado fruits treated with chitosan

**Figure 3:**
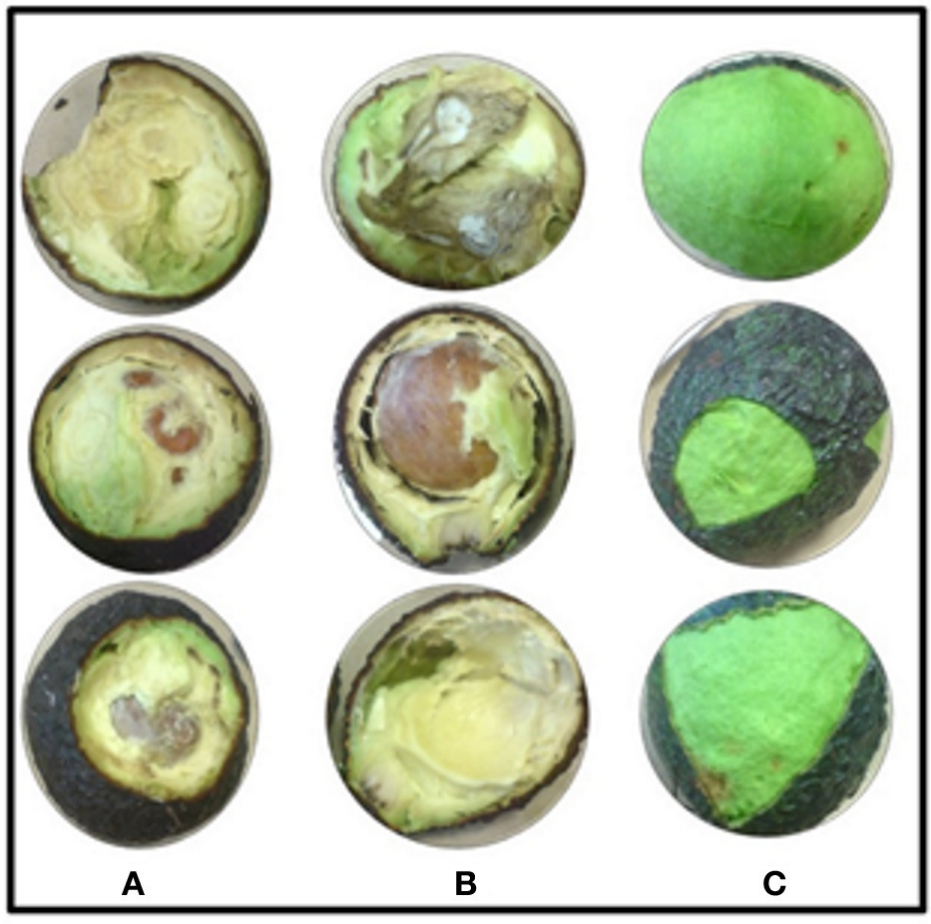
Avocado fruit maturity at the intermediate state, within 7 days of treatment. **(A)** Control fruits (without chitosan, not-inoculated). **(B)** Inoculated fruits without chitosan. **(C)** Inoculated fruits and treated with Chitosan. Symptoms in **(A,B)** belong to anthracnose, (softening pulp and characteristic mycelium development of *C. gloeosporioides)*, whereas in **(C)** fruits treated with chitosan, do not present development of *C. gloeosporioides*.

Most control fruits developed anthracnose symptoms after 7 days, such as fleshiness and softening pulp (Figure 3A), indicating that C. *gloeosporioides* was present in its latent state. When fruits were inoculated with 40 mL of a suspension of 1 × 10^6^ spores/mL, all of them developed anthracnose and it was observed the growth and invasion of the pathogen covering most of the fruit, showing more drastic symptoms than control fruits (Figure 3B). In contrast, only 10% of fruits inoculated with *C. gloeosporioides* and treated with LMWC showed deterioration in quality and the pathogen failed to spread, maintaining a healthy fruit (Figure 3C). These results indicate that LMWC treatment readily increases the avocado fruit resistance against *C. gloeosporioides*.

### RNA-seq and mapping to the reference transcriptomes

To obtain and analyze the global transcript profile of the avocado fruit upon treatment/inoculation with chitosan/*C. gloeosporioides*, RNA samples were obtained from fruits treated with chitosan (Q condition), inoculated with the fungus (P condition), chitosan-treated and inoculated with the fungus (QP condition) and a control (non-treated and non-inoculated). The RNA from each condition at different set times, was used for cDNA library preparation and sequenced using the ILLUMINA platform HiSeq 2000. Over 300 million sequences were obtained with an average length of 100 bp in paired-end format. Table 1 shows the number of paired-end reads obtained for each of the treatments. Read alignment to the TAD (Transcriptome Avocado var Drymifolia), resulted in an average of 93% mapped reads, while alignment to the TFH (Transcriptome Fruit var Hass) resulted in an average of 83% sequencing reads mapped. However, mapping to the TFH resulted in an increase of reads aligned concordantly exactly 1 time (ACE = 1) (an average of 62%) whereas mapping to the TAD only an average of 18% of reads ACE = 1 were obtained (Table 1). Reads aligned concordantly exactly 1 time were used to avoid redundancy in further expression analysis (Langmead and Salzberg, 2012). These mapping differences are possibly due to the different characteristics of the two reference transcriptomes used here, TAD was derived from several organs of the drymifolia avocado plant (including fruit) while TFH was generated from the Hass avocado fruit only (Ibarra-Laclette et al., 2015; Kilaru et al., 2015).

**Table 1:** Comparison of the mapped of sequences^a^ of different treatments using two transcriptomes of *Persea americana*: avocado drymifolia (TAD) and avocado Hass (TFH).

**Condition**	**Time (h)**	**Total Reads**	^b^**ACE=1**		^c^**ACE>1**		**% Overall alignment**	
		**Paired-end**	**TAD**	**TFH**	**TAD**	**TFH**	**TAD**	**TFH**
	0	20,631,843	21.9	60.9	67.1	21.5	92.9	89.7
Control fruits	1	15,265,622	19.5	60.7	66.8	21.3	93.4	91
	6/9	16,360,548	18.3	60.4	67.1	19.4	92.4	88.8
	24	21,012,239	17	64.6	71.1	17.5	94.1	90.4
Inoculated (P)	0	26,558,994	18.6	62.4	71	19.5	94	89.1
	1	40,574,501	16.9	61.2	68	17.4	92.9	88.9
	6/9	18,767,327	17.9	61.7	70	19.7	94.4	90
	24	21,095,442	17.6	63.2	71.8	19.6	94.6	90.4
Chitosan treatment (Q)	0	16,596,262	17.2	70.7	69.9	10.3	94.5	90.9
	1	17,946,164	18.2	58.5	64.6	17.4	91.2	86.9
	6/9	15,299,619	20.8	60.8	68	21.3	93.2	89.2
	24	13,715,619	19.7	60.4	67.7	19.4	92.7	87.8
Chitosan treatment and inoculated (QP)	0	15,457,171	17.3	61.3	68.4	17.7	92.9	88.4
	1	16,702,260	19	61.4	67.4	18.8	92.7	88.6
	6/9	18,244,858	19.6	61.6	70.3	21.1	93.8	89
	24	18,901,106	14.1	63.7	72.8	13.5	90.4	84.4

a *Values of ACE = 1, ACE > 1 and overall alignment represent the percentage of alignment using Bowtie2 program, P < 0.05*.

b *Aligned concordantly exactly 1 time*.

c *Aligned concordantly >1 times*.

### Differential gene expression profiles and gene ontology categorization

The differential gene expression profiles were obtained to identify those genes in the avocado fruit that were up- or down-regulated during treatment with chitosan or/and inoculation with the pathogen. Each of the sequenced libraries showed a similar pattern of the expression profiles using the two reference transcript datasets (TAD and TFH) (Table 2). The results show that, when compared with control fruits (non-treated and non-inoculated), at 1 h in the condition P (inoculated vs. control) and Q (treated with LMWC vs. control) the highest amount of unigenes with differential expression is obtained, whereas in QP (inoculated and treated with LMWC vs. control) the highest amount is observed at 24 h. In contrast, the minimum amount of differentially expressed genes was obtained in conditions P_0 and P_6/9, whereas in conditions Q and QP the minimum amount was at time 6/9. Considering all differentially expressed genes, up-regulated and down-regulated, using the two reference transcriptomes, we found that at the QP condition a higher differential expression was obtained compared to the control fruit, while in condition P a smaller amount of differentially expressed unigenes was obtained.

**Table 2:** Differential expression profile^a^ of different treatments using two reference transcriptomes of *Persea americana*: var drymifolia (TAD) and avocado Hass (TFH).

**Comparison matrix**	**Condition_time**	^b^**Down-Regulated**		**Up- regulated**		^c^**TDE**	
		**TAD**	**TFH**	**TAD**	**TFH**	**TAD**	**TFH**
Inoculated (P) vs. control fruit	P_0	27	15	240	154	1,888	1,422
	P_1	296	258	1,139	912		
	P_6/9	20	9	21	19		
	P_24	233	141	295	179		
Inoculated (P) and chitosan treatment (Q) vs. control fruit	QP_0	172	178	901	755	3,031	3,112
	QP_1	63	70	314	267		
	QP_6/9	16	17	70	53		
	QP_24	187	187	2,130	2,302		
Chitosan treatment (Q) vs. control fruit	Q_0	129	143	1,372	1,126	2,880	2,587
	Q_1	359	355	1,838	1,689		
	Q_6/9	5	8	52	53		
	Q_24	86	66	56	67		

a *Values represent log_2_(Fold Change), p_value < 0.05*.

b *Log_2_(FC) < −2 are Down-Regulated unigenes and Log_2_(FC) > +2 are Up-Regulated unigenes*.

c *TDE: Total differential expression for each comparison. Up and Down regulated unigenes*.

**Figure 4:**
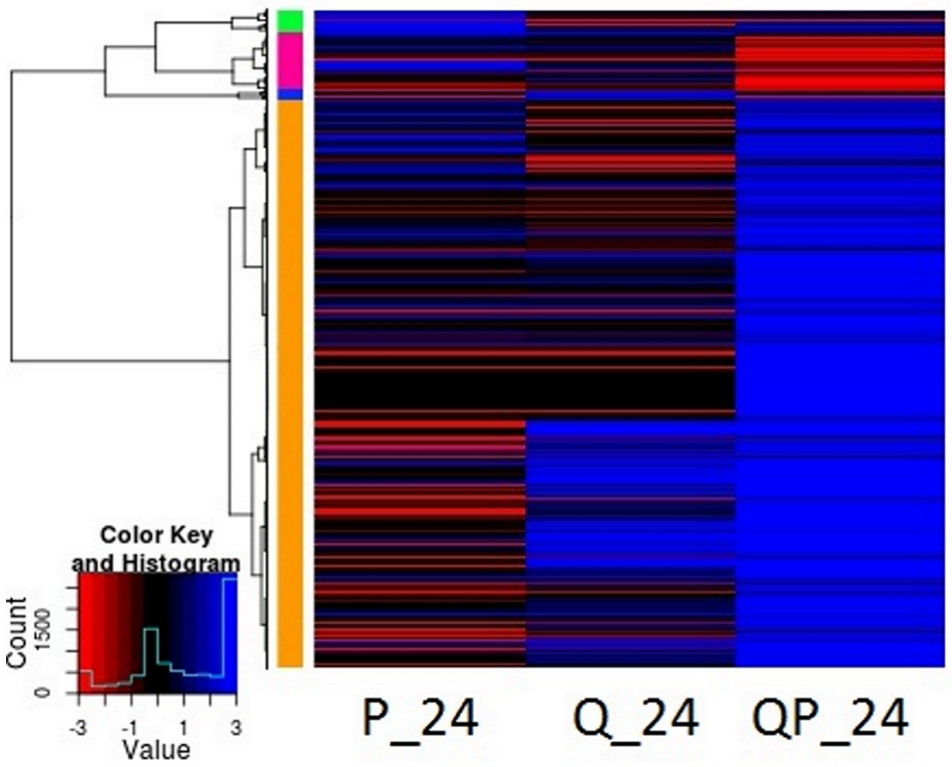
Heatmap representation of differentially expressed genes in P, Q, and QP conditions at 24 h post infection. Results are shown from mapping with avocado Hass transcriptome. Each line represents a differentially expressed gene. Red color represents down-regulated genes, blue color up-regulated and black color no differential expression gene. Most genes of the QP condition were mostly induced. HeatMap with R statistical program was used.

**Figure 5:**
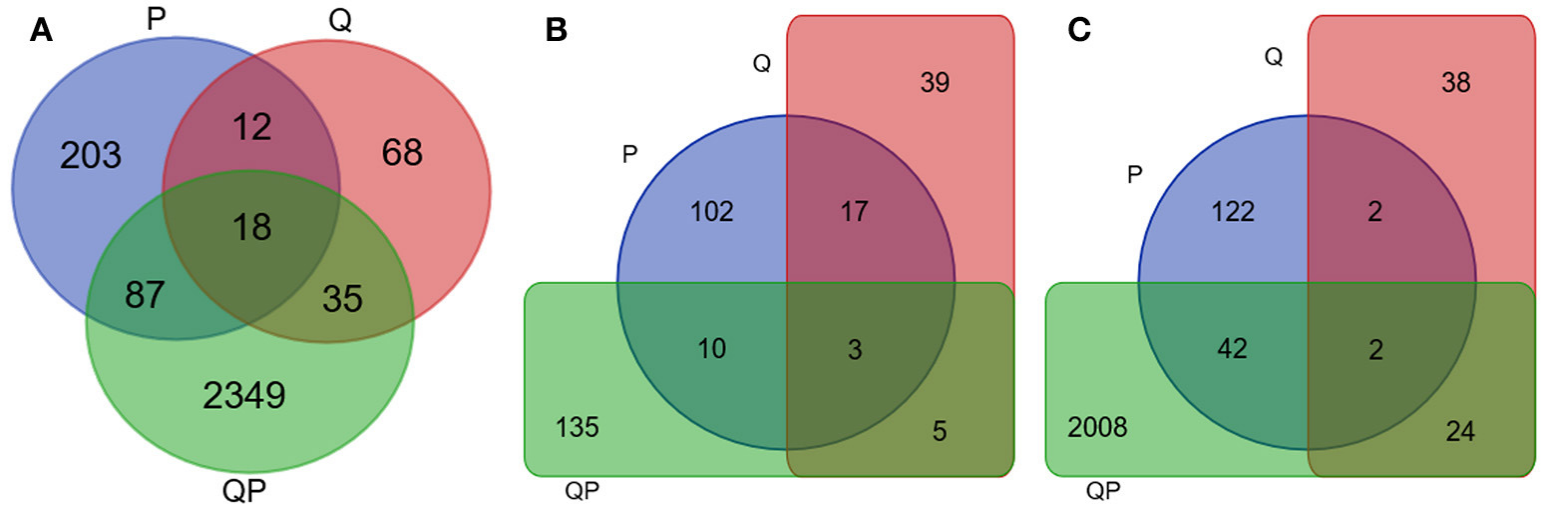
Venn diagram of differentially expressed genes in P (blue), Q (Red), and QP (green) conditions at 24 h. Results from mapping with avocado Hass transcriptome. **(A)** Up- and down-regulated unigenes. **(B)** Down-regulated unigenes. **(C)** Up-regulated unigenes.

Gene expression analysis at different sampling times shows that at 24 h most genes of the QP condition were induced (FC > +2) (Figure 4), whereas in the P condition were mostly repressed and in the Q condition the number of genes induced and repressed is similar. More than 2400 genes were differentially expressed in the QP_24 condition, from this set of genes 2000 were induced (Figure 5). Interestingly, the highest number of differential gene expressed was observed in Q condition at initial times (0 and 1 h), in contrast we observed that in the same Q condition there was a minor number of differential expressed genes at long time (24 h) in comparison with P and QP conditions. This suggests that the fruit induced its defense system by regulating several genes in short times just after treatment with chitosan.

To identify and assign enriched Gene Ontology (GO) terms to our dataset (Plant MetGenMap, Ontology Process, multi-test correction FDR, *p*_value < 0.05), all of the differentially up- and down-regulated unigenes showing sequence homology to *A. thaliana* (DNA sequences defined in terms by at least 60% similarity) were functionally categorized in biological process (BP), cellular component (CC), and molecular function (MF) (Table S3). The gene expression profile from the different conditions, that were analyzed by GO categorization showed important differences. In the P_24 condition some down-regulated unigenes were associated with “Systemic acquired resistance (SAR)” and “incompatible interaction with fungus,” suggesting that a decrease in the expression of these genes could be the cause of the observed disease development in the fruit. Other unigenes that are up-regulated in the same condition (P) are associated with “Defense response to fungus,” but such defense is not effective since the fruit is susceptible in this condition. In contrast, when only chitosan is present (condition Q_24) some of the unigenes that are categorized in biotic stimulus, specifically those involved in SAR response, are up-regulated. The QP_24 condition shows a possible combined response between the processes occurring in both Q and P separately, for example, the unigenes UN26087 (Log_2_FC = −4.37 and Log_2_FC = −3.17, P and QP conditions, respectively) and UN17145 (Log_2_FC = 3.09 and Log_2_FC = 5.30, Q and QP conditions, respectively), are both associated with SAR. Interestingly, in the QP condition a considerable increase of the expression was observed in the genes that are classified in this category (Table 3). GO analysis of the QP condition at 24 h showed processes as response to stimulus, response to stress, response to chemical stimulus and response to abiotic stimulus as the main processes that are affected due to treatment with chitosan and pathogen inoculation (Table 4, and Table S4).

**Table 3:** GO Functional Classification of several up- and down- regulated unigenes from treatments at 24 h that participate in the GO category “Biological process: Response to abiotic or biotic stimulus” (*p*_value < 0.05).

**Id_unigen^a^**	**Locus_At**	**Sim^b^**	**Description GO^c^**	**Log_2_FC**
**P_24**				
UN22478	AT2G47730	54	Defense response to bacterium	3.84
UN24431	AT4G20970	59	Defense response to fungus	4.28
UN25204	AT4G37870	94	Defense response to fungus, incompatible interaction	−3.76
UN05540	AT1G11580	64	Response to bacterium	4.06
UN09181	AT3G45140	80	Response to bacterium	4.27
UN15753	AT3G16770	69	Response to other organism	−3.95
UN10274	AT1G20030	81	Response to other organism	3.53
UN33841	AT4G36000	71	Response to other organism	3.84
UN18390	AT3G28480	86	Systemic acquired resistance	−4.07
UN08200	AT3G24503	78	Systemic acquired resistance	−2.70
UN24647	AT1G43800	85	Systemic acquired resistance, salicylic acid mediated signaling pathway	−4.98
UN26087	AT2G38290	59	Systemic acquired resistance, salicylic acid mediated signaling pathway	−4.37
**Q_24**				
UN25204	AT4G37870	94	Defense response to fungus, incompatible interaction	−2.82
UN09181	AT3G45140	80	Response to bacterium	4.50
UN26952	AT3G02260	77	Response to fungus	4.92
UN02048	AT5G42020	47	Response to heat	2.87
UN33302	AT3G06490	59	Response to salt stress	−5.04
UN10235	AT5G13930	77	Response to UV	−3.74
UN17145	AT3G02550	64	Systemic acquired resistance, salicylic acid mediated signaling pathway	3.09
UN41590	AT1G15520	79	Systemic acquired resistance, salicylic acid mediated signaling pathway	7.48
**Qp_24**				
UN22478	AT2G47730	54	Defense response to bacterium	4.51
UN41690	AT1G28480	54	Defense response to bacterium	4.75
UN61218	AT2G35930	60	Defense response to fungus	−5.30
UN22722	AT2G15890	48	Defense response to fungus	7.03
UN00578	ATCG00480	82	Defense response to fungus, incompatible interaction	3.59
UN09181	AT3G45140	80	Response to bacterium	4.64
UN49784	AT3G62550	69	Response to molecule of fungal origin	5.12
UN02593	AT1G33440	79	Response to nematode	−3.22
UN15753	AT3G16770	69	Response to other organism	3.27
UN39665	AT5G17760	68	Systemic acquired resistance	−2.46
UN26087	AT2G38290	59	Systemic acquired resistance, salicylic acid mediated signaling pathway	−3.17
UN24647	AT1G43800	85	Systemic acquired resistance, salicylic acid mediated signaling pathway	2.55
UN27548	AT3G03000	84	Systemic acquired resistance, salicylic acid mediated signaling pathway	3.06
UN06422	AT4G13510	66	Systemic acquired resistance, salicylic acid mediated signaling pathway	3.46
UN20428	AT4G09650	53	Systemic acquired resistance, salicylic acid mediated signaling pathway	3.56
UN67382	AT1G74360	73	Systemic acquired resistance, salicylic acid mediated signaling pathway	3.62
UN17145	AT3G02550	64	Systemic acquired resistance, salicylic acid mediated signaling pathway	5.30

a *Contig: transcriptome avocado var drymifolia*.

b *% Similarity between avocado transcripts (TAD) and A.thaliana (Blast of TAD vs. A.thaliana genome)*.

c *Description SuperViewer GO: (http://bar.utoronto.ca/ntools/cgi-bin/ntools_classification_superviewer.cgi)*.

**Table 4:** Top 10 enriched GO terms of QP_24. Terms from GO (*p*-value < 0.05) Up-regulated (530 unigenes) and Down-Regulated (79 unigenes).

**Up-regulated**			**Down-Regulated**		
**Gene ontology term**	**Number of unigenes**	**Cluster frequency (%)**	**Gene Ontology term**	**Number of unigenes**	**Cluster frequency (%)**
Response to stimulus	91	17.20	Response to stimulus	15	19.00
Response to stress	61	11.50	Transport	13	16.50
Response to chemical stimulus	45	8.50	Establishment of localization	13	16.50
Response to abiotic stimulus	31	5.80	Localization	13	16.50
Response to temperature stimulus	19	3.60	Response to chemical stimulus	10	12.70
Response to other organism	17	3.20	Response to endogenous stimulus	9	11.40
Response to heat	10	1.90	Post-translational protein modification	9	11.40
Response to jasmonic acid stimulus	10	1.90	Response to hormone stimulus	8	10.10
Response to wounding	9	1.70	Anatomical structure development	8	10.10
Response to carbohydrate stimulus	8	1.50	Protein amino acid phosphorylation	8	10.10

### Metabolic pathways related to biotic stress

**Figure 6:**
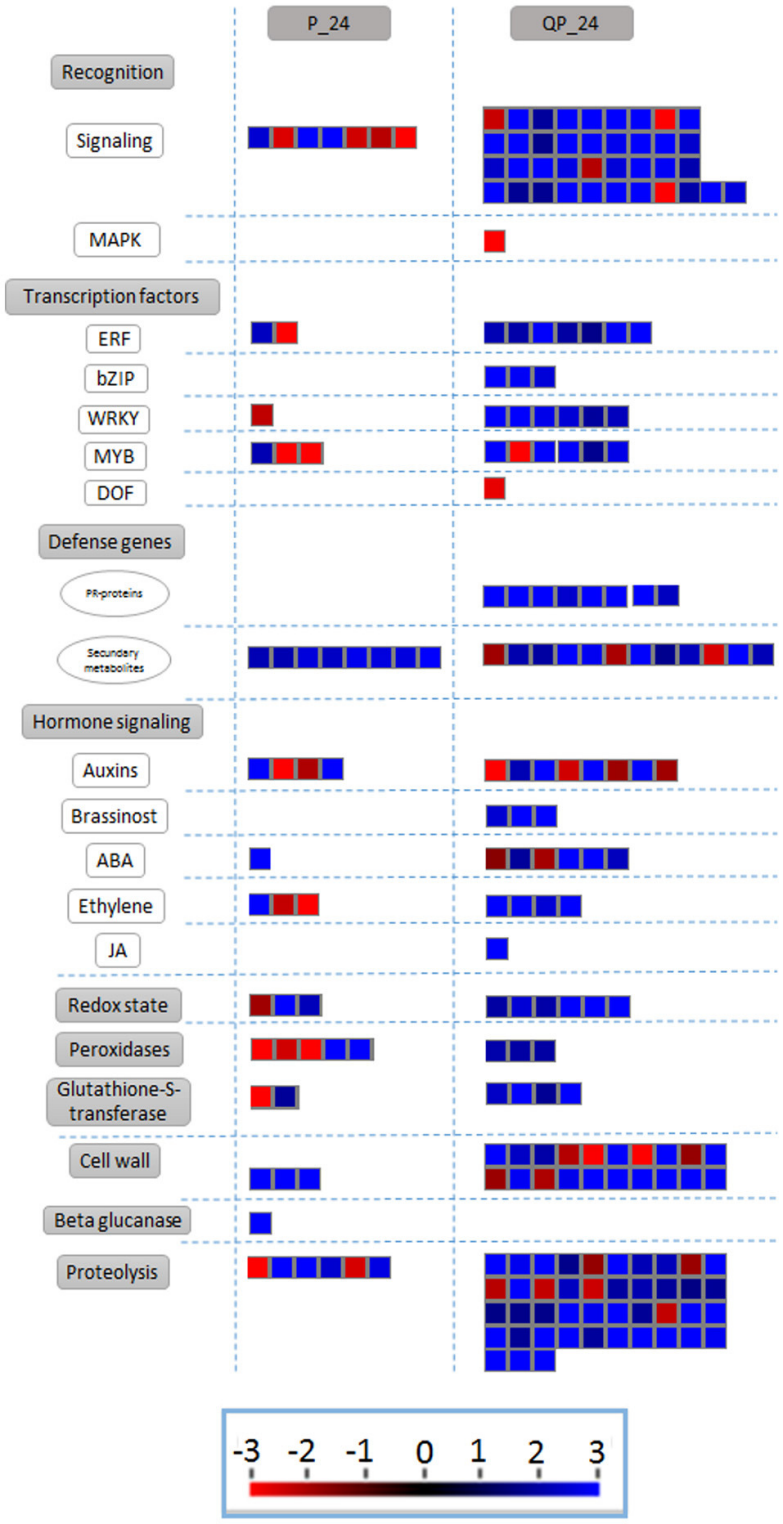
Differentially expressed transcripts related to biotic stress responses. The fold change of gene expression levels were analyzed using MapMan. Small red and blue squares represent up- and down-regulated genes, respectively.

In order to analyze changes in metabolic pathways related to biotic stress in the fruit for each of the treatments, we used the MapMan software (Usadel et al., 2009) based on the homology to *A. thaliana* genes of known function. Several subsets of genes encoding receptor-like kinases, signaling proteins, transcriptional factors, oxidative stress response elements, secondary metabolism factors, phytohormone-responsive genes and defense-related genes were identified. Notably, a greater number of genes were differentially expressed in the QP condition in all of the identified metabolic pathways. As described before, 24 h after inoculation fruits without chitosan showed characteristic symptoms of infection caused by *C. gloeosporioides* (Figure 2), whereas in chitosan treated fruits infection was not visible and a large number of genes related response to different metabolic processes were induced, mainly genes related to biotic stress responses (Figure 6).

An increase in the expression of unigenes actively involved in metabolic pathways related to biotic stress is observed from early times in fruits treated with LMW chitosan (Q_0) (Table 5). GO classification shows the relationship of some induced unigenes related to SAR and those involved in the salicylic acid and jasmonic acid signaling pathways. Also, a slight induction of genes involved in response to both biotic and abiotic stress is observed, suggesting that a priming state could be acting where the fruit is conditioned for the super-activation of defenses activated by pathogen inoculation.

**Table 5:** GO functional classification of unigenes related to “response to biotic stimulus” in fruits treated with chitosan at initial time (Q_0).

**Avocado unigene^a^Contig ID**	**^b^AT Locus ID**	**^c^Log_2_ (FC)**	**^d^Gene Ontology**	**^d^Description**
**Q_0**				
PA10010937	At1g73500	1.91	GO:0009651	Response to salt stress
PA10001010	At5g17310	2.0	GO:0009651	Response to salt stress
PA10000034	At3g15353	2.0	GO:0009651	Response to salt stress
UN28089	At3g04720	1.78	GO:0009627	Systemic acquired resistance
PA10034792	At2g23810	1.87	GO:0009612	Response to mechanical stimulus
PA10005805	At5g42050	2.08	GO:0009409	Response to cold
PA10022314	At3g04120	1.63	GO:0006972	Hyperosmotic response
PA10003223	At2g22240	1.67	GO:0042742	Defense response to bacterium
PA10024501	At1g32640	2.07	GO:2000068	Regulation of defense response to insect
**Q_1**				
PA10007344	At5g43060	1.95	GO:0009651	Response to salt stress
PA10010937	At1g73500	1.98	GO:0009651	Response to salt stress
PA10001010	At5g17310	2.05	GO:0009651	Response to salt stress
PA10012376	At5g20250	1.77	GO:0009416	Response to light stimulus
PA10008863	At2g26710	1.97	GO:0009416	Response to light stimulus
PA10009014	At1g68050	1.93	GO:0009637	Response to blue light
UN27548	At3g03000	1.86	GO:0009862	Systemic acquired resistance, salicylic acid mediated signaling pathway
PA10001012	At3g12490	1.72	GO:0009414	Response to water deprivation
UN11494	At3g46620	2.10	GO:0009414	Response to water deprivation
PA10007478	At5g59550	2.02	GO:0009414	Response to water deprivation
PA10022147	At4g24240	1.95	GO:0009408	Response to heat
PA10001322	At3g48990	1.61	GO:0050832	Defense response to fungus
UN20644	At2g35980	1.94	GO:0050832	Defense response to fungus
PA10020372	At3g12500	2.07	GO:0050832	Defense response to fungus
PA10005138	At3g05880	2.07	GO:0009266	Response to temperature stimulus
PA10006492	At2g20990	1.80	GO:0009409	Response to cold
PA10000793	At1g47128	1.56	GO:0006972	Hyperosmotic response
PA10015059	At2g22240	1.78	GO:0042742	Defense response to bacterium
UN01018	At1g55020	2.01	GO:0009816	Defense response to bacterium, incompatible interaction
PA10002905	At3g05550	1.67	GO:0001666	Response to hypoxia
PA10008712	At1g17290	1.82	GO:0001666	Response to hypoxia

a *Contig TAD: UNXXXX; Contig TFH: PA1XXXXXX*.

b *Locus ID A. thaliana gene homology with TAD and TFH unigenes*.

c *Log_2_ (FC), P_value < 0.05*.

d *Classification SuperViewer Tool w/Bootstrap of locus A. thaliana*.

### Gene expression validation by qRT-PCR

To validate and confirm the gene expression results obtained by bioinformatics analysis of our RNA-seq data, we performed real time quantitative RT-PCR of several avocado unigenes that showed different expression patterns (Table 6). For this, we considered unigenes that mapped to both reference transcriptomes and that corresponded to various metabolic pathways such as ethylene, secondary metabolites, transcription factors and pathogenesis related. The results show a trend of expression as well as a high correlation between the expression results by RNA-seq and qRT-PCR.

**Table 6:** Analysis of gene expression and validation by qRT-PCR of several unigenes mapped to Hass transcriptome (TFH) and drymifolia transcriptome (TAD) (*P* < 0.05).

**Contig ID^a^**	**Name**	**Sample**	**^b^RNA-seq**	**^c^qRT-PCR**
PA10053717	WRKY22	P_1	4.79	2.9
		QP_24	4.63	6.9
		Q_1	5.54	4.45
PA10048435	Avfae1	P_1	8	2.36
		P_24	−4.14	−2.2
		Q_1	7.16	3.89
		QP_1	8.41	6.84
PA10000364	Avfad12-3	P_1	1.96	1.62
		Q_0	1.64	0.89
PA10002664	CHS	Q_1	−2.14	−1.03
PA10079021	NB-arc	P_1	3.12	3.59
UN37342	WRKY22	P_1	4.91	2.9
		Q_1	5.75	4.45
		QP_24	4.71	6.9
UN69179	Avfae1	P_1	11.43	2.36
		P_24	−3.87	−2.2
		Q_1	10.98	3.89
		QP_1	11.87	6.84
UN02803	Avfad12-3	P_1	2.52	1.62
		Q_0	1.61	0.89
UN29560	ERF	P_1	1.91	5.26
		Q_0	2.63	4.25
UN30532	4-CL	Q_0	3.16	4.99
UN59130	NB-arc	P_1	4.07	3.59
		Q_1	4.23	1.16
UN27536	Avox	QP_24	10.31	2.58
UN42782	CHS	Q_1	−2.58	−1.03
UN28880	FLS	Q_1	−2.61	−4.18

a *Contig TAD: UNXXXX; Contig TFH: PA1XXXXXX*.

b *Log2 (Fold Change)*.

c *Log2 (RQ)*.

## Discussion

### RNA sequencing and differential expression profile

The use of two reference transcriptomes (TDH and TFH) allowed us to have a greater coverage in the analysis of our sequencing data. The comparative analysis of our data with both reference transcriptomes resulted in different percentage of mapped sequences (Table 1). On average 50% less of differentially expressed unigenes (induced and repressed) were obtained in the P condition when compared with those obtained in Q and QP conditions. On the other hand, the little difference of genes differentially expressed in the P condition in comparison with the control, could be explained by the fact that the control fruits are frequently naturally infected with the phytopathogen *C. gloeosporioides*, which is commonly found in the host from early stages of fruit development (Beno-Moualem and Prusky, 2000; Mendgen and Hahn, 2002). Thus, the development of the infection can be observed as the fruit ripens. However, in our experimental setting, we observed that at 1 h post inoculation, the fruit is highly resistant probably due to the presence of antifungal compounds (Table 2) (Yakoby et al., 2002; Wharton and Diéguez-Uribeondo, 2004; Prusky and Lichter, 2007). By contrast, in the presence of chitosan and the pathogen (QP), there is a strong defense response even at 24 h post-inoculation (Table 2). We hypothesize that the observed differences between the expression profile in QP condition in comparison with Q condition, are possibly due to potentiation of cellular signaling processes, as a consequence of the increased stress in the fruit induced by the chitosan-pathogen-fruit interaction.

The major GO categories for the P condition were the “response to stress to abiotic or biotic stimulus” and “metabolic processes,” whereas in the Q condition there were “cellular processes” and “response to stress to abiotic or biotic stimulus.” Interestingly, when the fruit was inoculated with the pathogen and treated with chitosan (QP), a significant increase of differentially expressed genes that were located in several GO categories was observed, for example, “cellular processes,” “metabolic processes,” “response to stress abiotic or biotic stimulus,” “biological processes,” “transport,” “cell organization and biogenesis” and “signal transduction,” this indicate that the chitosan could be able to activate different metabolic pathways involved in the defense of the fruit.

In order to have specific annotation data from the profile expression obtained here, we used the information from *A. thaliana* based on sequence homology. Several avocado unigenes that were involved in SAR and that were induced in the P, Q, and QP conditions, have homologs in *A. thaliana* genes involved in the response to different pathogens. For example, AT3G02550 (leucine-rich repeat receptor–like kinase NILR1) and AT1G74360 (nematode-induced LRR-RLK 1) are required for induction of innate immunity to parasitic nematode (Mendy et al., 2017); AT5G17760 (P-loop containing nucleoside triphosphate hydrolases superfamily) is related to plant response during geminivirus infection (Ascencio-Ibáñez et al., 2008); AT1G15520 (ABCG40 atp-binding cassette g40) is related to *Phytophthora* resistance (Wang et al., 2015); and AT3G02260 (Calossin-like protein required for polar auxin transport) is involved in immune response to *H. arabidopsidis* (Meteignier et al., 2017) (Table 3). This suggests that the chitosan and/or phytopathogen perception could converge in some signal points activating common metabolic pathways in the fruit. Although different defense responses may be activated depending on the tissue or organ attacked by a pathogen, some common pathways could be present and similarly efficient to stop an infection, however, more studies are needed to shed light on the defense mechanisms induced by elicitors like chitosan in fruits.

The analysis to identify enriched GO terms (Table 4) showed the enrichment of various metabolic processes that could contribute to the resistance of the fruit against C. *gloeosporioides*. It has been reported that plants treated with chitosan present mostly biochemical and molecular changes that include: chromatin alterations (Hartney et al., 2007; Hadwiger, 2008), increases in cytosolic Ca2+ (Zuppini et al., 2004), activation of MAP-kinases (Yin et al., 2009), oxidative burst (Paulert et al., 2010), callose deposition (Kohle et al., 1985), increase in pathogenesis-related (PR) gene mRNA, PR protein synthesis (Berumen-Varela et al., 2015), phytoalexin accumulation, hypersensitive response (HR) (Hadwiger, 2013), and in some systems, synthesis of jasmonic acid (JA) and abscisic acid (ABA) and accumulation of hydrogen peroxide (Lin et al., 2005; Iriti and Faoro, 2009). Recently, the use of next-generation sequencing has allowed different studies that identify global changes in cellular processes that are affected by both biotic and abiotic stress. For example, Coqueiro et al. (2015) identified by RNA-seq different processes that were affected by chitosan treatments in citrus leaves. Here we performed RNA-Seq analysis in avocado fruit treated/inoculated with chitosan/*C. gloeosporioides* to identify different metabolic pathways and processes involved in the induced resistance in the fruit.

### Activation of the defense-priming state

It has been reported that activation of the defense-priming state is accompanied by processes such as systemic acquired resistance (Jung et al., 2009) and induced systemic resistance (Pieterse et al., 2014), and also by wound-induced resistance (Chassot et al., 2008). Activation of these processes in short times possibly induces the “state of priming” related to sensitization of a cell or organism for enhanced defense; this condition causes faster and more robust activation of defense responses upon challenge with a pathogen (Conrath et al., 2015). Our results suggest that chitosan could induce a state of priming in short times after application, which promotes effective fruit resistance against C. *gloeosporioides* (Table 5).

On the other hand, several studies (Wang et al., 2010; Rodríguez-Carpena et al., 2011; Widsten et al., 2014) indicate that there are compounds in the epicarp of avocado fruits, mainly phenolic compounds such as catechins, epicatechin, proanthocyanidins, and quercetin, which have antibacterial and antifungal activity, and a high antioxidant capacity. It has also been identified the diene AFD (1-acetoxy-2-hidroxiy-4-oxo-heneicosa 12, 16 diene) (Ardi et al., 1998) with high antifungal capacity. Our analysis showed that treatment of fruits with chitosan downregulates some genes involved in biosynthesis of phenylpropanoids (CHS and FLS) (Table 6) whereas the gene encoding to 4-Coumarate coenzyme A ligase (4CL) is up-regulated. Likewise, we found transcription factors such as WRKY22 (Povero et al., 2011) and ERF (Oñate-Sánchez and Singh, 2002; Vallejo-Reyna et al., 2015) that were induced in different treatments with chitosan compared to control treatments in avocado fruits. According with our results, the genes involved in the biosynthesis of diene AFD (Avfad1 and Avfae12-3) are up-regulated in the fruits treated with chitosan (Table 6), suggesting a direct association between the diene AFD synthesis and chitosan.

The results presented in this study shows that chitosan acts as a molecule able to induce multiple metabolic responses in avocado fruit that collectively implements a defense system capable of counteract the infection by *C. gloeosporioides*. However, further studies are needed to experimentally determine the role and function of up- and down-regulated genes in fruits treated with chitosan and to dissect their participation in the resistance to pathogens as *C. gloesporioides* in avocado.

## Author contributions

AC-L provided the idea of the work. AC-L and LX designed the experiments. PG and UL contributed with the *in vivo* assays in fruit. EC performed the identification of the phytopathogen. LX, SG, and JV performed the bioinformatics analysis. LH contributed in the sequencing and generation of libraries. LX and JV performed qRT-PCR assays. LX, AC-L, JV, and LH participated in the interpretation of results and critically reviewed the manuscript. LX wrote the paper. All authors read and approved the final manuscript.

### Conflict of interest statement

The authors declare that the research was conducted in the absence of any commercial or financial relationships that could be construed as a potential conflict of interest. The reviewer OVL declared a shared affiliation, though no other collaboration, with one of the authors JVA to the handling Editor, who ensured that the process met the standards of a fair and objective review.
