# Supplementary material for: Transcriptomic Analysis of Avocado Hass (Persea americana Mill) in the Interaction System Fruit-Chitosan-Colletotrichum
Source: Front Plant Sci. 2017 Jun 8;8:956. doi: 10.3389/fpls.2017.00956 (PMC5462954; doi:10.3389/fpls.2017.00956)
Supplement: Supplementary file 2 [file DataSheet2.docx]

Additional File 2. *Molecular identification of isolates of Colletotrichum sp*


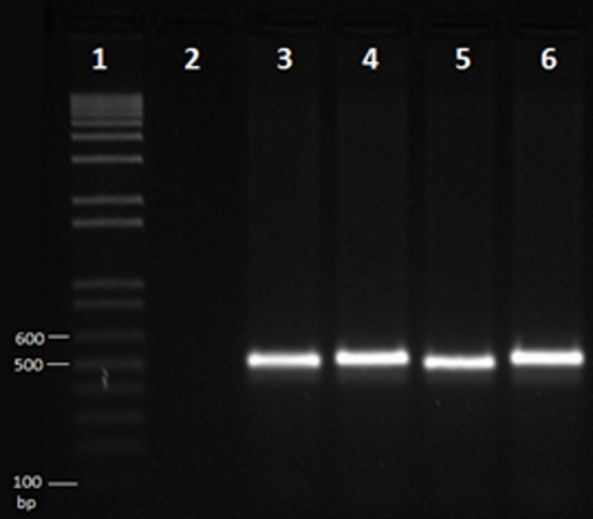


Figure A2. Amplification of the ITS region of *Colletotrichum* sp (ColAG1) isolated from avocado with symptoms of anthracnose, with oligonucleotides ITS1 and ITS 4. Lane 1; Molecular weight marker 1Kb. Lane 2; negative control (without DNA). Lines 3-6 amplified ITS fragment 560 bp, of 4 strains of the same isolated *Colletotrichum* obtained from strain-monosporic.

*Table A2.1; Sequencing^1^ amplification product ITS1 and ITS4.*

| >ColAG1 |
| --- |
| TACGCTTTATTAACACTACACCAAACCCCTGTGAACATACCAATTGTTGCCTCGGCGGATCAGCCCGCTCCCGGTAAAACGGGACGGCCCGCCAGAGGACCCCTAAACTCTGTTTCTATATGTAACTTCTGAGTAAAACCATAAATAAATCAAAACTTTCAACAACGGATCTCTTGGTTCTGGCATCGATGAAGAACGCAGCAAAATGCGATAAGTAATGTGAATTGCAGAATTCAGTGAATCATCGAATCTTTGAACGCACATTGCGCCCGCCAGTATTCTGGCGGGCATGCCTGTTCGAGCGTCATTTCAACCCTCAAGCCCAGCTTGGTGTTGGGGCACGTTTTGATTTATTTATGGTTTTACTCGGAAGTTAGATATATAAAGAAATTTTAAGGGTCTTCTGGAGCCCAGCCCTTTTTAAACGACAGCGGGCTGACGGCCAAGGCAACATTGGAATGTTGACGGGGATTGGGAATTGTTAACCGGTGAACGTTGCTCTCAATTTCGGGAGGAGGTATCCATTAGCGGAGGAGCACTCCAATACTGGGAACATACAGTTGTTGCCTGGCGGATCAGGCTCCGGTAAAAGGGACGGCCGCCGAGACCCTAAACTCTGTTTCTATATGTAACTTTCTGAGTAAAACCATAAATAAATCAAACTTTCAACAACGAATCTCTTGGTTCTGGCATCATGAAGAACGCACAAAATGCCATAAGCTAATGTGAATTGCAAATTCAGTGAATCATCGAATCTTTGAACGCACATTGCGCCCGCCATATTCTGGCGGGGCATGCTGTTCAGCGTCTTTTCCCCCCTCCCCTTTGGGTGTGGGGACTCCGCAATCAAACGGTTTCAAATTTAATTGGCGGGCACCTCAACTTCCTAAGTTAATAATAAAACCCCCTTTACCGGTTAACCTCCCGG |

^1^The sequencing service was requested to the company Humanizing Geomics Macrogen (Seoul, Korea)

Table A2.2. Blast results^1^, Sequence^2^ producing significant alignment. Max score (577) and identity of 99%.

| Range 1: 11 to 331 | | | | |
| --- | --- | --- | --- | --- |
| **Score** | **Expect** | **Identities** | **Gaps** | **Strand** |
| 577 bits(312) | 1e-163 | 318/321(99%) | 0/321(0%) | Plus/Plus |

LSC-15 18 ACACCAAACCCCTGTGAACATACCAATTGTTGCCTCGGCGGATCAGCCCGCTCCCGGTAA 77

|| || ||||||||||||||||||||||||||||||||||||||||||||||||||||||

ColAG1 11 ACTCCCAACCCCTGTGAACATACCAATTGTTGCCTCGGCGGATCAGCCCGCTCCCGGTAA 70

LSC-15 78 AACGGGACGGCCCGCCAGAGGACCCCTAAACTCTGTTTCTATATGTAACTTCTGAGTAAA 137

||||||||||||||||||||||||||||||||||||||||||||||||||||||||||||

ColAG1 71 AACGGGACGGCCCGCCAGAGGACCCCTAAACTCTGTTTCTATATGTAACTTCTGAGTAAA 130

LSC-15 138 ACCATAAATAAATCAAAACTTTCAACAACGGATCTCTTGGTTCTGGCATCGATGAAGAAC 197

||||||||||||||||||||||||||||||||||||||||||||||||||||||||||||

ColAG1 131 ACCATAAATAAATCAAAACTTTCAACAACGGATCTCTTGGTTCTGGCATCGATGAAGAAC 190

LSC-15 198 GCAGCAAAATGCGATAAGTAATGTGAATTGCAGAATTCAGTGAATCATCGAATCTTTGAA 257

||||||||||||||||||||||||||||||||||||||||||||||||||||||||||||

ColAG1 191 GCAGCAAAATGCGATAAGTAATGTGAATTGCAGAATTCAGTGAATCATCGAATCTTTGAA 250

LSC-15 258 CGCACATTGCGCCCGCCAGTATTCTGGCGGGCATGCCTGTTCGAGCGTCATTTCAACCCT 317

||||||||||||||||||||||||||||||||||||||||||||||||||||||||||||

ColAG1 251 CGCACATTGCGCCCGCCAGTATTCTGGCGGGCATGCCTGTTCGAGCGTCATTTCAACCCT 310

LSC-15 318 CAAGCCCAGCTTGGTGTTGGG 338

||||| |||||||||||||||

ColAG1 311 CAAGCACAGCTTGGTGTTGGG 331

| Range 2: 22 to 414h #2 | | | | |
| --- | --- | --- | --- | --- |
| **Score** | **Expect** | **Identities** | **Gaps** | **Strand** |
| 368 bits(199) | 9e-101 | 338/399(85%) | 34/399(8%) | Plus/Plus |

LSC-15 547 CTGGGAACATA-CAGTTGTTGCCT-GGCGGATCAG---GCT-CCGGTAAAA-GGGACGG- 598

||| ||||||| || ||||||||| |||||||||| ||| ||||||||| |||||||

ColAG1 22 CTGTGAACATACCAATTGTTGCCTCGGCGGATCAGCCCGCTCCCGGTAAAACGGGACGGC 81

LSC-15 599 CCGCC-GA-GA-CCCTAAACTCTGTTTCTATATGTAACTTTCTGAGTAAAACCATAAATA 655

||||| || || |||||||||||||||||||||||||| |||||||||||||||||||||

ColAG1 82 CCGCCAGAGGACCCCTAAACTCTGTTTCTATATGTAAC-TTCTGAGTAAAACCATAAATA 140

LSC-15 656 AATC-AAACTTTCAACAACGAATCTCTTGGTTCTGGCATC-ATGAAGAACGCA-CAAAAT 712

|||| ||||||||||||||| ||||||||||||||||||| |||||||||||| ||||||

ColAG1 141 AATCAAAACTTTCAACAACGGATCTCTTGGTTCTGGCATCGATGAAGAACGCAGCAAAAT 200

LSC-15 713 GCCATAAGCTAATGTGAATTGCA-AATTCAGTGAATCATCGAATCTTTGAACGCACATTG 771

|| ||||| |||||||||||||| ||||||||||||||||||||||||||||||||||||

ColAG1 201 GCGATAAG-TAATGTGAATTGCAGAATTCAGTGAATCATCGAATCTTTGAACGCACATTG 259

LSC-15 772 CGCCCGCCA-TATTCTGGCGGGGCATGC-TGTTC-AGCGTCTTTTCCCCCCTC---C-C- 823

||||||||| |||||||||||| ||||| ||||| |||||| |||| ||||| | |

ColAG1 260 CGCCCGCCAGTATTCTGGCGGG-CATGCCTGTTCGAGCGTCATTTCAACCCTCAAGCACA 318

LSC-15 824 CTTTGGGTGTGGGGACTCCGCAATCAAACG-GTT--TCAAATTTAATTGGCGGGCACCTC 880

|||| ||| ||||||| || | | || ||| ||||||| |||||||| ||| ||

ColAG1 319 GCTTGG-TGTTGGGACTC-GCGTTAATTCGCGTTCCTCAAATTG-ATTGGCGGTCACGTC 375

LSC-15 881 AA-CTTCC-TAAGTTAATAATAAAACCCCCTTTACCGGT 917

| ||||| || || || |||||||| | |||| |||

ColAG1 376 GAGCTTCCATAGCGTAGTAGTAAAACCCTCGTTACTGGT 414

^1^ Blastn: <https://blast.ncbi.nlm.nih.gov>. (Database: nr, Nucleotide collection (nt), Organism *Colletotrichum gloeosporioides* (taxid:474922)

2. GenBank: KU097215.1. ACCESSION: KU097215 REGION: 11..331. *Colletotrichum gloeosporioides* isolate LSC-15 internal transcribed spacer 1, partial sequence; 5.8S ribosomal RNA gene and internal transcribed spacer 2, complete sequence; and 28S ribosomal RNA gene, partial sequence
