# Supplementary material for: Transcriptomic Analysis of Avocado Hass (Persea americana Mill) in the Interaction System Fruit-Chitosan-Colletotrichum
Source: Front Plant Sci. 2017 Jun 8;8:956. doi: 10.3389/fpls.2017.00956 (PMC5462954; doi:10.3389/fpls.2017.00956)
Supplement: Supplementary file 5 [file DataSheet5.docx]

Supplementary Material

**TRANSCRIPTOMIC ANALYSIS OF AVOCADO HASS FRUIT (*Persea americana* Mill) IN THE INTERACTION SYSTEM AVOCADO FRUIT-CHITOSAN-*Colletotrichum***

Luis-Angel Xoca-Orozco^1^, Angélica Cuellar-Torres^1^, Sandra González-Morales^2^, Porfirio Gutiérrez-Martínez^1^, Ulises López-García^1^, Luis Herrera-Estrella^2*^, Julio Vega-Arreguín^3*^, Alejandra Chacón-López^1*^.

**Correspondence**

Julio Vega-Arreguín: [jvega.arreguin@gmail.com](mailto:jvega.arreguin@gmail.com)

Luis Herrera-Estrella: [lherrera@langebio.cinvestav.mx](mailto:lherrera@langebio.cinvestav.mx)

Alejandra Chacón-López: [alei.chacon@gmail.com](mailto:alei.chacon@gmail.com)

# Supplementary Materials

**Supplemental Table S1**: Primer used for validation expression in qPCR

**Supplemental File S2**: Comparison of conserved regions of *C. gloeosporioides* with amplified ITS

**Supplement Table S3:** Functional Classification SuperViewer GO of up- and down-regulated unigenes of all treatments

**Supplemental Table S4**: Enriched GO terms all conditions, using Plant MetGenMap, Ontology Process, multi-test correction FDR, p_value < 0.05
